# Uptake and outcomes of a novel community‐based HIV post‐exposure prophylaxis (PEP) programme in rural Kenya and Uganda

**DOI:** 10.1002/jia2.25670

**Published:** 2021-06-21

**Authors:** James Ayieko, Maya L Petersen, Jane Kabami, Florence Mwangwa, Fred Opel, Marilyn Nyabuti, Edwin D Charlebois, James Peng, Catherine A Koss, Laura B Balzer, Gabriel Chamie, Elizabeth A Bukusi, Moses R Kamya, Diane V Havlir

**Affiliations:** ^1^ Center for Microbiology Research Kenya Medical Research Institute Nairobi Kenya; ^2^ Department of Biostatistics University of California Berkeley CA USA; ^3^ Infectious Diseases Research Collaboration Kampala Uganda; ^4^ Department of Medicine University of California San Francisco CA USA; ^5^ Department of Biostatistics and Epidemiology University of Massachusetts Amherst MA USA; ^6^ Department of Medicine Makerere University Kampala Uganda

**Keywords:** post‐exposure prophylaxis (PEP), pre‐exposure prophylaxis (PrEP), HIV prevention, implementation, uptake, high‐risk exposure

## Abstract

**Introduction:**

Antiretroviral‐based HIV prevention, including pre‐exposure prophylaxis (PrEP), is expanding in generalized epidemic settings, but additional prevention options are needed for individuals with periodic, high‐risk sexual exposures. Non‐occupational post‐exposure prophylaxis (PEP) is recommended in global guidelines. However, in Africa, awareness of and access to PEP for sexual exposures are limited. We assessed feasibility, acceptability, uptake and adherence in a pilot study of a patient‐centred PEP programme with options for facility‐ or community‐based service delivery.

**Methods:**

After population‐level HIV testing with universal access to PrEP for persons at elevated HIV risk (SEARCH Trial:NCT01864603), we conducted a pilot PEP study in five rural communities in Kenya and Uganda between December 2018 and May 2019. We assessed barriers to PEP in the population and implemented an intervention to address these barriers, building on existing in‐country PEP protocols. We used community leaders for sensitization. Test kits and medications were acquired through the Ministry of Health supply chain and healthcare providers based at the Ministry of Health clinics were trained on PEP delivery. Additional intervention components were (a)PEP availability seven days/week, (b)PEP hotline staffed by providers and (c)option for out‐of‐facility medication delivery. We assessed implementation using the Proctor framework and measured seroconversions via repeat HIV testing. Successful “PEP completion” was defined as self‐reported adherence over four weeks of therapy with post‐PEP HIV testing.

**Results:**

Community leaders were able to sensitize and mobilize for PEP. The Ministry of Health supplied test kits and PEP medications; after training, healthcare providers delivered the 28‐day regimen with high completion rates. Among 124 persons who sought PEP, 66% were female, 24% were ≤25 years and 42% were fisherfolk. Of these, 20% reported exposure with a serodifferent partner, 72% with a new or existing relationship and 7% from transactional sex. 12% of all visits were conducted at out‐of‐facility community‐based sites; 35% of participants had ≥1 out‐of‐facility visit. No serious adverse events were reported. Overall, 85% met the definition of PEP completion. There were no HIV seroconversions.

**Conclusions:**

Among individuals with elevated‐risk exposures in rural East African communities, patient‐centred PEP was feasible, acceptable and provides a promising addition to the current prevention toolkit.

## 1 Introduction

HIV post‐exposure prophylaxis (PEP), the use of antiretroviral medications for 28 days to prevent HIV acquisition after high‐risk exposure, has long been available, and is recommended in World Health Organization guidelines [1, 2]. However, it has not routinely been integrated in the prevention toolkit in sub‐Saharan Africa (SSA) beyond limited use restricted to occupational risks among healthcare workers, female sex workers and men who have sex with men [3, 4]. Some reasons for PEP underutilization include widespread unawareness of this prevention option, fear of misuse limiting offers of PEP, lack of provider training in screening for eligible candidates and stigma and discrimination against high‐risk groups such as sex workers and men who have sex with men [3, 5, 6, 7]. Pre‐exposure prophylaxis (PrEP) is increasing, but not reaching all populations at risk, thus additional prevention measures are needed [8, 9]. We therefore sought to assess feasibility, acceptability, uptake and adherence in rural Kenyan and Ugandan settings in a pilot study of a patient‐centred PEP programme with options for facility‐ or community‐based service delivery.

## 2 Methods

### 2.1 Study setting

Between December 2018 and May 2019, we conducted a pilot study of PEP delivery for high‐risk HIV exposures in five rural communities in Kenya and Uganda within the SEARCH HIV test‐and treat trial described previously (NCT01864603) [10]. The study offered universal access to PrEP during population‐level HIV testing to persons at elevated HIV risk (based on serodifferent partnership, an empiric risk score or self‐assessment of risk [11, 12]. In five study communities, we conducted PEP pilot to provide an additional prevention option following elevated‐risk HIV exposures.

### 2.2 Study procedures

We assessed barriers to PEP within the study communities using focus groups with community members and healthcare providers in order to inform PEP intervention implementation. Our assessment of barriers to PEP uptake identified the following: 1) lack of community awareness about PEP as a prevention option including where to access the services; 2) lack of health system level requests for PEP drugs and HIV test kits to accommodate PEP beyond occupational exposure, and lack of flexibility in visit hours and location of service delivery for PEP; 3) lack of confidence among healthcare providers to prescribe and deliver PEP to the general population and, 4) concerns among clients regarding lack of confidentiality, side effects and lack of access to trained healthcare providers to answer client questions around PEP.

We then designed and implemented a multi‐component intervention to address these barriers, building on existing in‐country PEP protocols [13, 14]. (1) Community sensitization and mobilization around HIV prevention and the role of PEP was conducted using community leaders. (2) Healthcare providers were trained on screening for eligible participants, 28‐day antiretroviral‐PEP regimen administration and participant monitoring. Patient‐centred service provision and confidentiality were emphasized. (3) Requisition for supplies such as antiretrovirals and HIV test kits for PEP delivery were made through the usual Ministry of Health mechanisms. (4) Participants presenting themselves at study clinics for PEP were screened. Those found eligible were tested at baseline using country standard antibody‐based HIV testing, and if negative, were initiated on PEP with follow‐up visits either in person or via phone call at week 2 and a HIV test at week 4, 12 and 24. As per country guidelines, PEP regimens initially comprised atazanavir/ritonavir, lamivudine, tenofovir (ATV/r/3TC/TDF) and later, dolutegravir (DTG/3TC/TDF). Participants were counselled around HIV prevention at the study visits. Our PEP delivery pilot also comprised; (a) PEP availability seven days/week, (b) PEP mobile phone hotline (text or voice) staffed by providers, (c) option for out‐of‐facility community‐based medication delivery and (d) condom dispensation at study visits.

### 2.3 Implementation evaluation framework

We applied a modified version of the implementation evaluation framework proposed by Proctor et al. []. We defined four levels at which the PEP delivery intervention was designed to act, and for each, defined the actor (the target of the implementation intervention), the action (the implementation intervention), the target (the goal to be accomplished by the action), the measures used to assess fidelity (defined as whether the intervention was executed as planned) and the measures used to assess the implementation outcome (whether the intervention achieved its desired goal) (Table 1). The four levels at which the intervention was designed to act were (i) the community and opinion leaders, (ii) the healthcare providers, (iii) the Ministry of Health and clinics and (iv) the clients receiving PEP.

**Table 1:** Feasibility metrics and outcomes during the conduct of the PEP delivery pilot in five rural communities in Uganda and Kenya

Actor: Who are you trying to act on?	Action: Implementation intervention	Target: What are you trying to accomplish with action?	Fidelity Measurement: Was intervention done as planned?	Implementation Outcome: Did it accomplish intended goal?
Community leaders	Train community leaders on PEP in the community	Understand concept of PEP and explain to the community situations in which PEP would be indicated	Two meetings every month held with community leaders in each community for two months	Community leaders participated in sensitization of the community of PEP as a HIV prevention option
Ministry of health	Make commodity request for test kits and medication in a timely manner to ensure uninterrupted supply Obtain guideline booklets on PEP (regimen, adverse events) for providers	Ensure availability of PEP medications and HIV test kits at clinics Ensure guidelines on PEP for reference are available for healthcare workers	Commodity requests made using the existing supply requisition system and commodities supplied Treatment guidelines availed in all clinics	158 bottles of regimen medications availed and dispensed over the period of the pilot study No stock outs of PEP occurred during the study period 343 HIV test kits used
Healthcare providers	Train health providers on identifying eligible participants for PEP and offering PEP in a high HIV prevalence setting, including patient confidentiality and patient education on side effects Train health providers on structured follow‐up visits designed to enhance adherence and regimen completion	Enhance competence and ability of healthcare providers to offer PEP to participants and conduct structured follow‐up visits including HIV tests	Trainings on PEP delivery conducted for healthcare providers at all targeted clinics	All health providers engaged (n = 15) were able to offer PEP to willing participants All 15 providers dispensed PEP to at least one participant during the study Providers completed 267 follow‐up visits for participants
Client/community	Offer PEP to eligible participants	Enhance PEP uptake and adherence to medication and follow‐up visits	PEP availed and offered at all clinics for those willing to initiate PEP and follow‐up visits conducted	124 participants were enrolled and started on PEP with 88% reporting adherence and 97% being retained at four weeks

### 2.4 Population and measures

PEP uptake was defined as enrolment into the pilot with receipt of a 28‐day course of antiretroviral drugs for PEP. We measured adherence by self‐report using three‐day recall. Adverse events in the pilot were measured using DAIDS scale [16]. Successful “PEP completion” was defined as self‐reported adherence over four weeks of therapy with post‐PEP HIV testing. We evaluated the proportions of participants who initiated PEP, were retained in the study, self‐reported adherence to PEP and received HIV testing at week 4, 12 and 24.

## 3 Results

### 3.1 Fidelity outcomes

Twelve community leaders comprising religious and administrative leaders from the five communities were identified and sensitized on PEP as an HIV prevention option. Four community meetings per clinic were attended by healthcare providers to train and sensitize the community leadership. Fifteen healthcare providers comprising seven clinical officers and eight nurses from six clinics were trained on the PEP delivery intervention at their respective clinics. The requisition for supply for drugs and test kits was made monthly through the Ministry of Health with monthly reports of commodities consumed being submitted through the health facility leadership.

### 3.2 Implementation outcomes

The community religious and administrative leaders demonstrated a good understanding of PEP as an HIV prevention option in high‐HIV prevalence settings and participated in successful sensitization and mobilization of the communities (Table 1). The governments through the Ministries of Health availed supplies on request such as antiretrovirals for PEP, HIV test kits and national treatment guidelines for reference. Healthcare providers in the PEP delivery clinics were trained on delivery of PEP to those reporting high‐HIV risk exposures. The providers were able to assess risk, conduct HIV testing and initiate eligible participants on PEP. They conducted follow‐up for up to 24 weeks while assessing adherence by self‐report for the duration participants were on PEP. Visits were conducted at clinics or out‐of‐facility based on participants’ preference. Providers were also able to reach participants on phone to monitor their progress, assess adherence and address any concerns such as side effects.

Community members with high‐HIV risk exposures willingly presented themselves to clinics offering PEP for clinician‐assessment and PEP initiation where applicable.

### 3.3 Uptake of PEP

A total of 124 participants were initiated on PEP in our pilot; 84 (68%) were from Kenya; 82 (66%) were male. Two‐thirds of the participants were aged between 15 and 35 years; 30 (24%) were ≤25 years; 52 (42%) were aged 25 to 34 years. 71 (58%) were married; 8 (7%) in polygamous marriages and 46 (37%) reported being single. Sixty‐six (54%) were in occupations associated with higher HIV‐risk, most of these, 52 (42%) being fisherfolk. Among all participants, 91 (73%) were initiated on DTG/3TC/TDF, whereas the rest were initiated on an Atazanavir/ritonavir‐based regimen.

Among those who were enrolled in the pilot, various reasons were reported for initiating PEP. 83 (67%) were in new or existing relationships with partners who they suspected to be HIV infected. Of note, 14 (11%) of the sexual partners feared to be infected were reported as fisherfolk. Additionally, 17 (14%) reported unplanned sexual exposure with a serodifferent spouse, 10 (8%) from transactional sex, 6 (5%) from sexual intercourse with a known HIV‐infected person and 2 (2%) from non‐consensual sex.

### 3.4 PEP visits, adherence and retention

**Figure 1:**
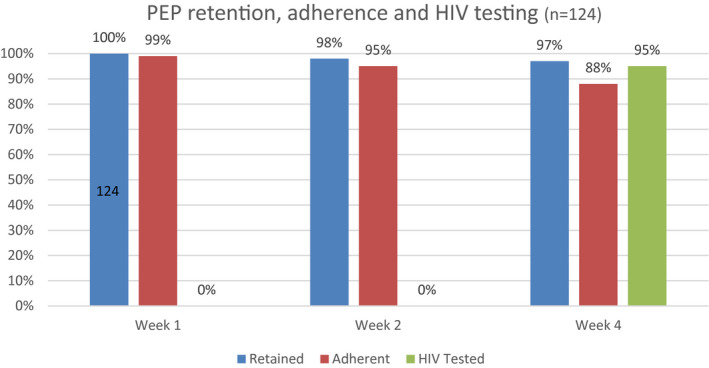
PEP retention, adherence and HIV testing during the course of the pilot. (Retention: Visit attendance at week 1, 2 and 4, Adherence: Use of PrEP measured by self‐report using three‐day recall, HIV Tested: Proportion receiving a HIV test at specified week 4 study visit).

Twelve percent of all visits were conducted at out‐of‐facility community locations; 43 (35%) of participants had ≥1 off‐site visit. 120 (97%) of participants were retained at a four‐week follow‐up with 109 (88%) reporting adherence over all follow‐up visits (Figure 1). Overall, 105 (85%) met the definition of PEP completion. 118 (95%) received HIV testing at week 4 follow‐up, 88% at week 12 and 83% at week 24. There were no HIV seroconversions.

### 3.5 Adverse events

No serious adverse events were reported. Two participants reported mild dizziness, two experienced mild generalized body malaise, one receiving atazanavir had grade two jaundice and one reported mild nausea. All events resolved spontaneously without having to stop PEP.

## 4 Discussion

Our study demonstrates feasibility of PEP as an HIV prevention option among the general population in rural sub‐Saharan Africa settings with high HIV prevalence. To our knowledge, this is the first documented study to target the general population in rural SSA with PEP for HIV prevention. In this pilot study, we found high retention and adherence to the 28‐day PEP regimen. Unlike other studies that have focused on evaluating PEP in known high‐risk groups such as sex workers and MSM [3, 6], we offered PEP to the general population within a high HIV prevalence setting. In these settings, individuals may not necessarily be on an ongoing continuous high‐risk exposure that would warrant PrEP [2]. However, they may experience sporadic or occasional unplanned episodes of high‐risk exposures that may end in infection. Judicious PEP use may be the prevention option of choice that will suppress new infections among this subgroup of the population. PEP as delivered in this pilot was shown to be safe and tolerable with no seroconversions observed.

PrEP has been shown to have high efficacy in randomized controlled trials among persons with high and sustained adherence [17]. However, many at risk either do not initiate PrEP when offered or have difficulties sustaining use over time [8, 18, 19]. Barriers to PrEP use include not feeling at risk, change in risk over time with the inability to anticipate or control, difficulties taking daily medication over a prolonged period, as well as stigma and fears of disclosure [18, 19]. PEP offers a complementary prevention measure to PrEP that addresses barriers such as unplanned episodes of high‐risk exposure, and shorter duration of use during a period when individuals are highly motivated to sustain use. Furthermore, PEP may provide a bridge to PrEP initiation or reengagement for some individuals; this potential merits further study in this context, including improved understanding of risk perception as a barrier to PrEP..

Components of our intervention designed to address barriers to PEP use reveal the need for a client‐centred approach. The high retention and adherence observed may be attributed to the flexibility in visit hours, choice of visit locations as well as phone access to a healthcare provider whenever required. One‐third of our participants opted for community‐based visits at least once in the course of follow‐up, demonstrating the value of flexibility and the potential to improve retention among enrolees in prevention interventions.

Limitations of our study include self‐report of PEP adherence which is subject to social desirability bias. However, no participants seroconverted during the trial, suggesting adequate drug levels to prevent infection. Second, our sample size was small and therefore meaningful comparison of data stratified by gender or region could not be carried out. Finally, the study started with protease inhibitor regimens (27% of clients), no longer the standard‐of‐care; however, current integrase inhibitor‐based regimens are more tolerable and would be expected to better facilitate uptake and adherence.

As an implementation outcome in the model proposed by Proctor et al.[15], sustainability is crucial. The existing commodity supply chain, healthcare staffing and community sensitization strategy have the capability to initiate PEP programmes and further improve the uptake of this prevention option. It will be important to do a formal cost analysis to inform scalability of this intervention. This set up could ensure long‐term viability by integrating the programme within existing policies and practices [20].

## 5 Conclusions

Among persons with high‐risk HIV exposures in a high prevalence, rural East African setting, a patient‐centred PEP programme was feasible, acceptable and provides a promising addition to the current prevention armamentarium.

## Competing Interests

All authors receive grant support from the NIH, Gilead Sciences provided Truvada for the SEARCH study, CAK has received grant support to institution from Gilead Research Scholars Program in HIV.

## Authors’ Contributions

All authors participated in the conduct of the study. JA, MP, DVH, FO, JK, FM, GC, MN, EDC, EAB, CAK and MRK designed and participated in the implementation of the study. JP, DVH, MP and LBB evaluated data integrity. JA, DVH and MP drafted the first version of the manuscript which was reviewed and endorsed by all authors.

## Ethical Approval

The study received ethical approvals from the University of California, San Francisco Committee on Human Research, Makerere University School of Medicine Research and Ethics Committee, the Ugandan National Council on Science and Technology (Uganda), and the Kenya Medical Research Institute Ethical Review Committee. All participants provided written informed consent in their preferred language prior to enrolment and participation in the study.
